# Associations Between Routinely Measured Biomarkers and Pancreatic Cancer Risk in Individuals with Type 2 Diabetes Pre- and Post-Diagnosis

**DOI:** 10.3390/cancers18091428

**Published:** 2026-04-30

**Authors:** Ziying Zhang, Sofia Carlsson, Niklas Hammar, Pilar Acedo, Beth Russell, Shahram Kordasti, Debashis Sarker, Mieke Van Hemelrijck, Aida Santaolalla

**Affiliations:** 1 Comprehensive Cancer Centre, School of Cancer and Pharmaceutical Sciences, KCL—King’s College London, London SE1 9RT, UK; 2 Institute of Environmental Medicine, Karolinska Institutet, 171 77 Stockholm, Sweden; 3 Institute for Liver and Digestive Health, Royal Free Hospital, UCL—University College London, London NW3 2QG, UK; 4 Transforming Cancer Outcomes Through Research (TOUR), KCL—King’s College London, London SE1 9RT, UK; beth.russell@kcl.ac.uk (B.R.);; 5 Haematology Department, Guy’s and St Thomas’ NHS Trust, London SE1 9RT, UK; 6 Comprehensive Cancer Centre, Guy’s Hospital, London SE1 9RT, UK; debashis.sarker@kcl.ac.uk

**Keywords:** pancreatic cancer, newly diagnosed type 2 diabetes, serum biomarkers, alkaline phosphatase, haptoglobin, creatinine, cohort study

## Abstract

**Background/Objectives:** Individuals with type 2 diabetes have an increased risk of pancreatic cancer (PC), yet early detection markers remain limited. We examined associations between routinely measured serum biomarkers and subsequent PC risk in this population. **Methods**: Using the Swedish AMORIS cohort, 101,051 individuals aged ≥50 years at diabetes diagnosis with newly diagnosed type 2 diabetes (1969–2019) were analysed. Ten biomarkers, including liver enzymes, inflammatory markers, renal function, and lipid profiles, were assessed. The primary analysis focused on measurements within ±1 year of the diabetes diagnosis (*n* = 13,190; Cohort 1). Sensitivity analyses considered broader windows: −3, −5, and −10 years before diagnosis to +1 year after, and from the diagnosis to the end of the follow-up (Cohorts 2–5). Multivariable Cox models adjusted for age and sex were applied. **Results**: During a mean follow-up of 15.9 years, 192 individuals developed PC. In primary analysis, higher alkaline phosphatase was associated with increased PC risk (HR: 1.16, 95% CI: 1.09–1.24 per 1 SD increase). Higher creatinine was associated with reduced PC risk (HR: 0.55, 95% CI: 0.36–0.83 per 1 SD increase), while haptoglobin (HR: 1.20, 95% CI: 0.97–1.49 per 1 SD increase) suggested a modest positive association. Patterns were consistent across sensitivity analyses. **Conclusions**: In individuals ≥ 50 years with newly diagnosed type 2 diabetes, higher ALP and lower creatinine were independently associated with increased PC risk before and after diabetes diagnosis, while haptoglobin showed a modest positive association. As these biomarkers are routine laboratory panels, their potential to support risk stratification in primary care requires further evaluation within established risk-prediction frameworks.

## Simple Summary

Pancreatic cancer is often diagnosed at a late stage, resulting in very poor survival. People newly diagnosed with type 2 diabetes are known to have a higher risk of developing this cancer, yet there are no simple tools to identify who among them is most at risk. This study used data from over 100,000 individuals in Sweden to examine whether routine blood test results measured around the time of diabetes diagnosis could help predict future pancreatic cancer risk. We found that higher levels of alkaline phosphatase and haptoglobin, along with lower levels of creatinine, were consistently associated with increased risk. Because these blood tests are already performed as part of standard clinical care, they could potentially be used to flag high-risk individuals for closer monitoring and earlier detection.

## 1. Background

Pancreatic cancer (PC) is a highly lethal malignancy and a leading cause of mortality worldwide. According to GLOBOCAN 2022, the global incidence rate of PC was 4.9 cases per 100,000 people, while Europe reported a higher rate of 7.8 cases per 100,000 people in the same year [1,2]. The incidence of PC is higher in males than in females, and it is commonly diagnosed in individuals aged around 70–75 years. Mortality rates closely mirror incidence, with age-standardised mortality rates of 4.6 per 100,000 globally and 7.3 per 100,000 in Europe. Pancreatic cancer has an extremely poor prognosis, with a five-year survival rate of 9% [3]. Imaging techniques such as computed tomography (CT) and Magnetic Resonance Imaging (MRI) have limited sensitivity for early-stage tumours, and while endoscopic ultrasound (EUS) is more sensitive, it is invasive and unsuitable for routine screening [4,5].

Several studies have demonstrated an association between newly diagnosed type 2 diabetes and an increased risk of PC. Across different countries and regions, approximately 1% of individuals with diabetes (aged ≥50 in most studies) develop PC within 3 years of diagnosis [6]. Hong et al. validated that the prevalence of PC in individuals with diabetes was higher than in the general population in Korea [7]. Diabetes was associated with a 2- to 7-fold increased risk of PC in the prospective study [8]. Therefore, our study targets individuals with newly diagnosed type 2 diabetes and investigates potential factors that may help to identify individuals at high risk of developing PC early.

There is an urgent need to explore blood-based biomarkers as non-invasive, accessible, and cost-effective tools for identifying individuals at high risk of developing PC, particularly among those with type 2 diabetes. Numerous biomarkers have been studied in diabetes and general populations for PC risk prediction, including cancer-associated markers, glycaemic indicators, liver enzymes, lipid profiles, and inflammatory markers [9]. PC risk prediction models have incorporated factors such as the age at diabetes diagnosis, BMI changes, blood glucose levels, medication use, and the symptoms like abdominal pain or jaundice. However, the PC predictive value of many routine blood and serum markers, such as aminotransferase (ALT), alkaline phosphatase (ALP), gamma-glutamyl transferase (GGT), haptoglobin (HP), creatinine, fructosamine, and total cholesterol (TC), remains unclear in people with diabetes.

This study aims to address these gaps by investigating the association between standard-of-care blood/serum biomarkers and the risk of developing PC in individuals with newly diagnosed type 2 diabetes. Ultimately, the broader aim is to inform the development of practical risk-stratification approaches that, with further validation, may help identify high-risk individuals in primary care settings.

## 2. Methods

### 2.1. Study Population

**Figure 1:**
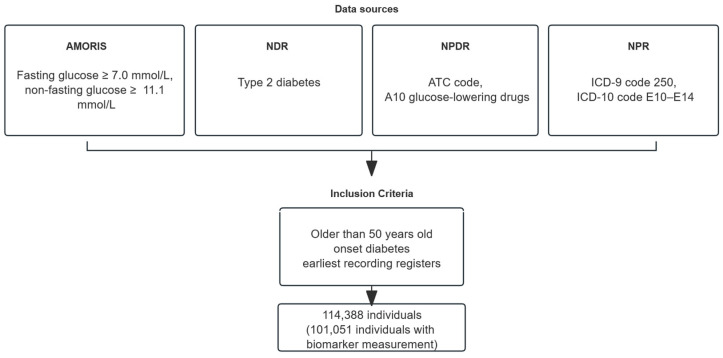
An algorithm for defining a diabetes population from four data sources. The data integration involves AMORIS (Apolipoprotein-related MOrtality RISk), NDR (National Diabetes Register), NPR (National Patient Register), and NPDR (National Prescribed Drug Register).

We conducted a prospective cohort study using the Swedish AMORIS database, which includes 812,073 individuals with comprehensive laboratory data linked to 24 national registries [10]. Participants with newly diagnosed type 2 diabetes between 1969 and 2019 who were aged ≥50 years at diagnosis were eligible. Diabetes diagnosis was defined using the earliest record across four sources (Figure 1): AMORIS biomarker data (fasting glucose ≥ 7.0 mmol/L or non-fasting ≥ 11.1 mmol/L), the National Diabetes Register (NDR), the National Patient Register (ICD-9 code 250; ICD-10 codes E10–E14), and the National Prescribed Drug Register (ATC code A10 for glucose-lowering medication). Individuals with prior pancreatic cancer, missing biomarker data, or PC diagnosis before the biomarker measurement were excluded.

### 2.2. Biomarker Assessment

**Figure 2:**
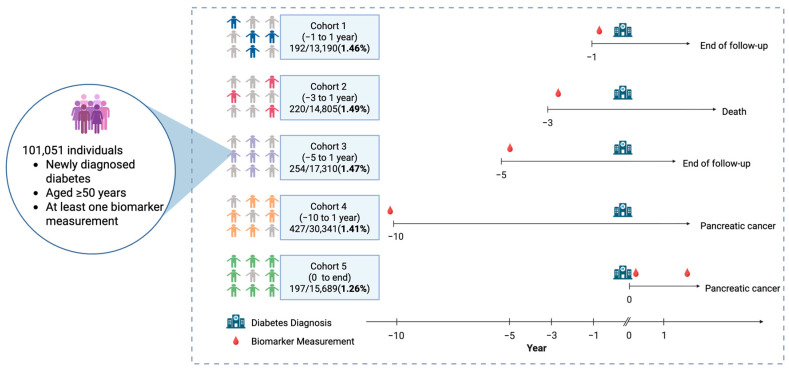
The cohort definition and the biomarker assessment windows for the pre-diagnostic and diagnostic periods for the several cohorts investigated. In each cohort, the grey figures represent the individuals who appear across all five cohorts. The coloured figures in each cohort represent the individuals who are newly included in that cohort compared to the previous one. For example, in Cohort 2 compared to Cohort 1, some individuals may not have data in the −1 to +1 year window but have biomarker measurements in the broader −3 to +1 year window. In this case, the red figures indicate people whose data was captured in the −3 to +1 year window but missing in the −1 to +1 year window. All the individuals included fall within the population, with 101,051 individuals with diabetes. The numbers are shown as “PC cases/total diabetes population in each cohort (percentage)”: Cohort 1, 192/13,190 (1.46%); Cohort 2, 220/14,805 (1.49%); Cohort 3, 254/17,310 (1.47%); Cohort 4, 427/30,341 (1.41%); and Cohort 5, 194/15,689 (1.26%). Each of the timelines constitutes examples of the patients’ pathways where the hospital icon marks the time of the diabetes diagnosis, and the blood drop icon indicates the first time a biomarker measurement was taken from the blood sample. The horizontal axis represents the time relative to the biomarker measurement window, extending toward the end of the follow-up period. The PC diagnosis was the primary outcome of interest in all five cohorts. The differing arrow lengths across the cohorts reflect the varying biomarker measurement windows used to define each cohort, not the differences in the outcome definition. The individuals who did not develop PC were censored at death, emigration, or the end of the follow-up (31 December 2019), whichever occurred first.

We analysed 10 routinely measured biomarkers: liver enzymes (alanine aminotransferase [ALT], alkaline phosphatase [ALP], gamma-glutamyl transferase [GGT]), inflammatory markers (haptoglobin, albumin), renal function (creatinine), lipid profile (total cholesterol [TC], triglycerides [TG]), and glycaemic indicators (fructosamine, fasting serum glucose). These biomarkers were selected based on (1) the routine availability in the AMORIS database, (2) prior evidence linking them to PC risk or related metabolic-inflammatory pathways in both general and populations with diabetes, and (3) representation across key biological domains (hepatic, renal, inflammatory, lipid, and glycaemic). All the biomarkers were analysed in the same laboratory (CALAB) using standardised procedures [10]. The primary analysis included measurements taken within ±1 year of the diabetes diagnosis, which constitutes Cohort 1. Sensitivity analyses considered four broader windows: −3, −5, and −10 years before the diagnosis to +1 year after, and from the diagnosis to the end of the follow-up, constituting Cohorts 2 to 5 (Figure 2).

### 2.3. Follow-Up and Outcome

The participants were followed from the diabetes diagnosis until the pancreatic cancer diagnosis, death, emigration, or the end of the follow-up (31 December 2019), whichever occurred first. Pancreatic cancer cases were identified from the Swedish Cancer Register using ICD-9 code 157 and ICD-10 code C25 [11].

### 2.4. Statistical Analysis

Descriptive statistics were presented using original values; continuous biomarkers were standardised (mean = 0, SD = 1) for inclusion in the regression analyses, and all the HRs therefore represent the association per 1 standard deviation (SD) increase in each biomarker. Multivariable Cox proportional hazards models adjusted for age and sex were used to estimate hazard ratios (HR) and 95% confidence intervals (CI) in the different models. All the biomarkers were included in the same model, and backward elimination (*p* < 0.20) identified independent predictors. The analyses adhered to the rule of ≥10 outcome events per predictor variable to reduce overfitting [12]. Statistical analyses were performed using SAS version 9.4 (SAS Institute, Cary, NC, USA). The proportional hazards assumption for all the Cox models was assessed using the supremum test based on cumulative martingale residuals, implemented via the *ASSESS PH*/*RESAMPLE* statement in *PROC PHREG* [13]; no significant violations were identified (all *p* > 0.05).

## 3. Results

We identified 101,051 individuals aged ≥50 years with newly diagnosed type 2 diabetes in the AMORIS cohort. The primary analysis that constitutes Cohort 1 included 13,190 individuals with biomarker measurements within ±1 year of the diabetes diagnosis; 192 (1.46%) developed PC during a mean follow-up of 15.9 years (Table 1).

### 3.1. Primary Cohort (−1 to +1 Year Window)

The results for univariable Cox models are presented in Table 2. In multivariable Cox models adjusted for age and sex, and after backward elimination, three biomarkers were retained as independent predictors. Higher ALP levels were associated with increased PC risk (HR: 1.16, 95% CI: 1.09–1.24 per 1 SD increase). Higher creatinine levels were associated with reduced PC risk (HR: 0.55, 95% CI: 0.36–0.83 per 1 SD increase). Haptoglobin suggested a modest positive association (HR: 1.20, 95% CI: 0.97–1.49 per 1 SD increase). The age at the diabetes diagnosis was also associated with risk (HR: 1.04, 95% CI: 1.01–1.08 per year). Other biomarkers, including ALT, albumin, GGT, cholesterol, triglycerides, fructosamine, and fasting glucose, showed no clear association in multivariable models (Table 3).

### 3.2. Sensitivity Analyses

Analyses across four broader time windows (−3, −5, −10 years to +1 year, and from the diagnosis to the end of the follow-up) yielded similar patterns. ALP remained consistently associated with increased risk (HR range: 1.14–1.17), creatinine retained its inverse association (HR range: 0.48–0.67), and haptoglobin showed hazard ratios between 1.19 and 1.23. These findings suggest that associations were stable across different pre-diagnostic and diagnostic periods (Table 3).

Overall, ALP demonstrated the most consistent association, while creatinine and haptoglobin were also retained in multivariable models. Differences in effect size between ALP and haptoglobin were small, and all the associations should be interpreted cautiously. Null findings for glucose and fructosamine indicate limited predictive value for glycaemic markers in this context.

## 4. Discussion

In this large cohort study of individuals aged ≥50 years with newly diagnosed type 2 diabetes, we observed higher ALP and modestly higher haptoglobin levels were associated with increased PC risk, while higher creatinine levels were associated with reduced PC risk. These associations persisted across multiple time windows, including periods up to 10 years prior to the diabetes diagnosis. While this cross-window stability is notable, our design does not permit causal inference; these findings therefore identify candidate biomarkers whose potential utility in risk stratification warrants further investigation in independent cohorts.

ALP demonstrated the most consistent association, with hazard ratios indicating a 14–17% increase in PC risk per SD increase across all the cohorts. This finding aligns with previous studies linking ALP to pancreatic cancer prediction, prognosis and survival [14,15,16,17,18,19,20,21,22]. Our study further extends this evidence by examining the associations in the pre-diagnostic phase among individuals with diabetes. Several mechanisms may explain this association. ALP is a well-established marker of hepatobiliary injury and cholestasis [23], and hepatobiliary disease itself is linked to increased PC risk [24]. Beyond direct biliary effects, elevated ALP may also reflect shared metabolic-inflammatory pathways—particularly insulin resistance and chronic inflammation—linking type 2 diabetes, MASLD, and pancreatic cancer [25]. Tumour cells may also directly up-regulate ALP via Wnt/β-catenin, BMP, and integrin signalling [21]. The hepatobiliary and metabolic-inflammatory pathways implicated above are amenable to modifiable lifestyle and metabolic interventions, such as weight management, physical activity, dietary modification, and glycaemic control, although whether targeted interventions on these pathways reduce PC risk requires prospective evaluation. Given that ALP is part of standard laboratory panels, its potential integration into risk prediction models warrants further investigation.

Creatinine showed an inverse association with PC risk (HR range: 0.48–0.67), which has rarely been emphasised in prior research. As serum creatinine reflects skeletal muscle mass [26,27], lower levels may indicate reduced muscle mass arising from frailty, sarcopenia, or other systemic conditions, and may not necessarily reflect a creatinine-specific relationship with pancreatic cancer. This is consistent with prospective evidence suggesting that low muscle mass is associated with higher pancreatic cancer risk [28]. This finding requires validation and exploration of longitudinal trends.

Haptoglobin, an acute-phase protein, suggested a modest positive association (HR range: 1.19–1.23), consistent with its role in systemic inflammation and prior evidence linking inflammatory markers to pancreatic carcinogenesis [29]. Hepatic haptoglobin synthesis may be induced by interleukin-6 (IL-6) and related cytokines through JAK/STAT3 signalling. IL-6 has also been implicated in pancreatic carcinogenesis, including pancreatic intraepithelial neoplasia, invasion and metastasis, through oncogenic signalling and modulation of the tumour immune microenvironment [30]. The consistent association across pre-diagnostic windows suggests that inflammatory processes may operate alongside the metabolic changes of type 2 diabetes.

Other biomarkers, including ALT, albumin, GGT, cholesterol, triglycerides, fructosamine, and fasting glucose, showed no clear association with PC risk after adjustment. These null findings are important, as prior studies have suggested potential links, particularly for glycaemic markers [31]. While prior literature has documented associations between glycaemic markers and PC risk, the absence of HbA1c limits the ability to fully evaluate the role of cumulative glycaemic exposure in our study. Differences may reflect the timing of measurement, population characteristics, or confounding adjustments.

A key strength of this study is that biomarker measurements were obtained as part of routine laboratory panels rather than targeted investigations, reducing selection bias. Additional strengths include the large sample size, long follow-up, and standardised laboratory procedures. Limitations include missing data for BMI, HbA1c, the latter of which may represent a more clinically relevant glycaemic indicator than fasting serum glucose in individuals with type 2 diabetes, smoking, and alcohol consumption. HbA1c data were available in the AMORIS database but were missing for a substantial proportion of participants, precluding its inclusion in the analyses. Future studies with more complete HbA1c data should examine its role as both a confounder and potential predictor of PC risk. Additionally, data on smoking and alcohol consumption, which are established PC risk factors and may represent unmeasured confounders, were incompletely recorded for a substantial proportion of participants. However, it is worth noting that several biomarkers included in this study, particularly GGT, ALT, and ALP, may serve as partial proxies for alcohol-related hepatic effects, thus partially mitigating this limitation. Furthermore, the study lacks genetic or epigenetic information [32], and the design also precludes causal inference. Our data did not allow differentiation between PDAC and other histological subtypes (e.g., pancreatic neuroendocrine tumours), as the limited number of PC events constrained subtype-specific analyses; restricting analyses to PDAC by excluding NETs would be preferable in future studies with other cohorts. Information on tumour location (pancreatic head versus body/tail) and the stage at diagnosis was also not available; subgroup analyses by these characteristics were therefore not feasible. Associations should therefore be interpreted cautiously and considered hypothesis-generating.

These present findings should be contextualised within existing pancreatic cancer screening and risk prediction frameworks. Current guidelines recommend surveillance only for high-risk individuals, such as those with hereditary predisposition or familial pancreatic cancer syndromes, and no universal screening programme exists for the general diabetic population. Established risk models such as the Enriching New-onset Diabetes for Pancreatic Cancer (END-PAC) score incorporate clinical variables including age, BMI change, and glucose trajectory to identify diabetic patients at elevated PC risk. The biomarkers identified in this present study, ALP, creatinine, and haptoglobin, are not currently included in these models, and their incremental predictive value remains to be evaluated. Future work should assess whether incorporating these routine biomarkers into existing risk models improves discrimination, before any role in primary-care surveillance can be considered. Additionally, the absence of BMI and HbA1c data in this present study represents a meaningful limitation; BMI in particular is an established PC risk factor and its omission may result in residual confounding, potentially affecting the magnitude of the observed associations.

Further research should validate these findings in independent cohorts, incorporate longitudinal biomarker trends, and integrate clinical and genetic factors into comprehensive risk prediction models. Given the accessibility of ALP, creatinine, and haptoglobin in routine care, their potential clinical utility in identifying high-risk individuals requires further evaluation within established risk-prediction frameworks.

## 5. Conclusions

In individuals ≥ 50 years with newly diagnosed type 2 diabetes, higher ALP and haptoglobin and lower creatinine were independently associated with increased PC risk in primary and sensitivity analysis. As these biomarkers are routine laboratory panels, they represent candidate predictors whose potential application in primary-care risk stratification requires validation in independent cohorts and integration into established risk-prediction frameworks.

## Figures and Tables

**Table 1:** The descriptive statistics of the study population Baseline demographic and the biomarker characteristics of the Primary cohort (Cohort 1) at the diabetes diagnosis, stratified by the PC development during the follow-up. Among 13,190 individuals with diabetes, 192 (1.46%) developed PC during a mean follow-up of 15.95 ± 9.13 years. fS, fasting serum. * *p* < 0.05.

		Individuals Without PC During Follow-Up N = 12,998 (98.54%)		Individuals with PC During Follow-Up N = 192 (1.46%)	*p*-Value
Characteristics	No. of Participants	Mean (SD) or %	No. of Participants	Mean (SD) or %	
Diabetes age, mean (SD)	12,998	63.37 (9.54)	192	62.55 (8.66)	0.231
Follow-up time, mean (SD)	12,998	15.95 (9.13)	192	9.27 (8.29)	<0.0001 *
Sex, %					0.66
Male	8228	63.30	125	65.10	
Female	4770	36.70	67	34.90	
Biomarkers, mean (SD)					
Alanine aminotransferase (ALT or Alat), U/L	10,548	40.97 (41.21)	162	46.89 (60.08)	0.235
ALBumin (ALB), g/L	10,108	42.03 (2.98)	154	42.17 (2.96)	0.575
Alkaline Phosphatase (ALP), µkat/L	9969	3.38 (1.80)	154	4.02 (5.07)	0.125
Gamma-Glutamyl Transferase (GGT), µkat/L	10,459	1.04 (1.79)	162	1.33 (2.81)	0.198
Haptoglobin, g/L	7377	1.22 (0.39)	118	1.22 (0.33)	0.892
Creatinine, μmol/L	11,128	88.67 (22.47)	162	85.17 (13.19)	0.001 *
Cholesterol (TC), mmol/L	11,109	6.11 (1.28)	169	5.81 (1.17)	0.000 *
Triglycerides (TG), mmol/L	11,083	2.36 (2.00)	168	2.06 (1.47)	0.011 *
Fructosamine, mmol/L	8284	2.66 (0.58)	129	2.67 (0.52)	0.837
fS-glucose, mmol/L	11,535	10.00 (3.78)	173	9.89 (3.42)	0.693

**Table 2:** Hazard ratio (HR) for risk of pancreatic cancer with 95% confidence intervals (CI) using univariable Cox proportional hazards models in five cohorts. All biomarkers, ALT, ALB, ALP, GGT, haptoglobin, creatinine, fS-glucose, fructosamine, TC, and TG, and age at diabetes diagnosis were initially analysed independently using univariable Cox-PH. Biomarker units: ALT (U/L), ALB (g/L), ALP (µkat/L), GGT (µkat/L), haptoglobin (g/L), creatinine (μmol/L), TC (mmol/L), TG (mmol/L), fructosamine (mmol/L), fS-glucose (mmol/L). All biomarkers were standardised (mean = 0, SD = 1) prior to analysis; hazard ratios represent the association per 1 SD increase.

Cohorts	−1 to 1 Years (Cohort 1)	−3 to 1 Years (Cohort 2)	−5 to 1 Years (Cohort 3)	−10 to 1 Years (Cohort 4)	0 to End (Cohort 5)
ALT	1.08 (0.99–1.17)	1.07 (0.97–1.17)	1.05 (0.95–1.16)	1.04 (0.97–1.12)	1.09 (1.01–1.17)
ALB	0.93 (0.79–1.10)	0.95 (0.81–1.11)	0.93 (0.80–1.08)	0.90 (0.80–1.01)	0.91 (0.77–1.07)
ALP	1.18 (1.13–1.24)	1.17 (1.12–1.23)	1.17 (1.11–1.23)	1.15 (1.10–1.20)	1.17 (1.11–1.24)
GGT	1.15 (1.05–1.27)	1.13 (1.03–1.24)	1.11 (1.00–1.22)	1.10 (1.01–1.19)	1.12 (1.01–1.24)
Haptoglobin	1.10 (0.92–1.32)	1.10 (0.92–1.31)	1.11 (0.94–1.31)	1.10 (0.96–1.27)	1.12 (0.94–1.34)
Creatinine	0.88 (0.70–1.10)	0.87 (0.68–1.11)	0.85 (0.67–1.07)	0.90 (0.76–1.05)	0.86 (0.68–1.09)
TC	0.75 (0.63–0.89)	0.76 (0.65–0.89)	0.79 (0.68–0.92)	0.82 (0.74–0.92)	0.75 (0.64–0.89)
TG	0.81 (0.65–1.01)	0.78 (0.63–0.97)	0.75 (0.62–0.92)	0.82 (0.71–0.95)	0.80 (0.65–0.99)
Fructosamine	1.02 (0.86–1.22)	0.96 (0.81–1.14)	0.92 (0.78–1.09)	0.90 (0.79–1.03)	1.05 (0.88–1.24)
fS-glucose	0.99 (0.86–1.16)	0.92 (0.79–1.08)	0.89 (0.77–1.03)	0.94 (0.84–1.06)	1.06 (0.91–1.22)
Age at diabetes diagnosis	1.03 (1.01–1.05)	1.03 (1.01–1.04)	1.03 (1.01–1.04)	1.02 (1.01–1.03)	1.02 (1.01–1.04)
Sex					
Male	1.00 (Ref)	1.00 (Ref)	1.00 (Ref)	1.00 (Ref)	1.00 (Ref)
Female	0.97 (0.72–1.30)	0.94 (0.72–1.25)	0.95 (0.74–1.23)	0.92 (0.76–1.12)	0.90 (0.67–1.20)

**Table 3:** The hazard ratio (HR) for the risk of pancreatic cancer with 95% confidence intervals (CI) using multivariate Cox proportional hazards models for all the biomarkers (ALT, ALB, ALP, GGT, haptoglobin, creatinine, TC, TG, fructosamine, fS-glucose) in five cohorts, adjusted for sex, age. The final models after backward elimination (*p* < 0.20) are also shown. A dash (‘–’) indicates that the biomarker was excluded during the backward elimination process. Biomarker units: ALT (U/L), ALB (g/L), ALP (µkat/L), GGT (µkat/L), haptoglobin (g/L), creatinine (μmol/L), TC (mmol/L), TG (mmol/L), fructosamine (mmol/L), fS-glucose (mmol/L). All the biomarkers were standardised (mean = 0, SD = 1) prior to analysis; the hazard ratios represent the association per 1 SD increase.

Cohorts	−1 to 1 Years (Cohort 1)	−3 to 1 Years (Cohort 2)	−5 to 1 Years (Cohort 3)	−10 to 1 Years (Cohort 4)	0 to End (Cohort 5)
Full model					
ALT	1.06 (0.95–1.18)	1.05 (0.94–1.18)	1.06 (0.94–1.19)	1.05 (0.93–1.18)	1.08 (0.98–1.19)
ALB	1.03 (0.80–1.33)	1.03 (0.81–1.32)	0.97 (0.77–1.22)	0.86 (0.71–1.05)	1.04 (0.81–1.34)
ALP	1.16 (1.09–1.25)	1.17 (1.09–1.25)	1.17 (1.09–1.25)	1.14 (1.08–1.21)	1.17 (1.09–1.26)
GGT	0.96 (0.72–1.30)	0.96 (0.71–1.29)	0.91 (0.67–1.25)	0.96 (0.75–1.22)	0.95 (0.71–1.27)
Haptoglobin	1.24 (0.99–1.54)	1.24 (1.00–1.53)	1.24 (1.02–1.52)	1.21 (1.02–1.44)	1.21 (0.98–1.51)
Creatinine	0.55 (0.36–0.84)	0.50 (0.31–0.82)	0.48 (0.31–0.76)	0.68 (0.49–0.95)	0.53 (0.34–0.83)
TC	0.84 (0.63–1.11)	0.84 (0.64–1.10)	0.92 (0.71–1.19)	0.97 (0.78–1.19)	0.84 (0.63–1.11)
TG	0.85 (0.60–1.19)	0.86 (0.62–1.18)	0.85 (0.63–1.15)	0.92 (0.74–1.14)	0.84 (0.60–1.18)
Fructosamine	1.14 (0.80–1.62)	1.13 (0.80–1.59)	0.93 (0.65–1.32)	1.08 (0.82–1.43)	1.01 (0.72–1.41)
fS-glucose	0.97 (0.66–1.42)	0.93 (0.64–1.35)	0.93 (0.65–1.32)	0.95 (0.71–1.29)	1.17 (0.83–1.65)
Age at diabetes diagnosis	1.04 (1.01–1.08)	1.04 (1.01–1.07)	1.04 (1.01–1.07)	1.03 (1.01–1.05)	1.05 (1.02–1.08)
Sex					
Male	1.00 (Ref)	1.00 (Ref)	1.00 (Ref)	1.00 (Ref)	1.00 (Ref)
Female	0.58 (0.34–1.01)	0.59 (0.35–1.00)	0.64 (0.39–1.05)	0.76 (0.50–1.15)	0.55 (0.32–0.94)
Final model following backward elimination *p* < 0.2					
ALT	-	-	-	-	1.07 (0.98–1.17)
ALB	-	-	-	0.87 (0.72–1.05)	-
ALP	1.16 (1.09–1.24)	1.16 (1.09–1.24)	1.17 (1.10–1.25)	1.14 (1.08–1.21)	1.16 (1.09–1.24)
Haptoglobin	1.20 (0.97–1.49)	1.21 (0.98–1.48)	1.23 (1.02–1.50)	1.19 (1.00–1.41)	1.20 (0.97–1.48)
Creatinine	0.55 (0.36–0.83)	0.50 (0.31–0.81)	0.48 (0.31–0.76)	0.67 (0.48–0.94)	0.53 (0.34–0.83)
TC	0.81 (0.63–1.04)	0.81 (0.64–1.04)	-	-	0.79 (0.62–1.02)
TG	-	-	0.82 (0.61–1.09)	-	-
Age at diabetes diagnosis	1.04 (1.01–1.08)	1.04 (1.01–1.07)	1.04 (1.01–1.07)	1.03 (1.01–1.05)	1.05 (1.02–1.08)
Sex					
Male	1.00 (Ref)	1.00 (Ref)	1.00 (Ref)	1.00 (Ref)	1.00 (Ref)
Female	0.59 (0.34–1.01)	0.59 (0.35–1.00)	0.62 (0.38–1.00)	0.75 (0.50–1.13)	0.56 (0.33–0.96)

## Data Availability

Access to data can be requested through the Access Committee of the AMORIS cohort, which is based in the Institute of Environmental Medicine at the Karolinska Institute.

## Abbreviations

The following abbreviations are used in this manuscript:

ALT	Alanine aminotransferase
ALB	Albumin
ALP	Alkaline phosphatase
AMORIS	Apolipoprotein-related MOrtality RISk
BMI	Body Mass Index
fS-Gluc	fasting serum glucose
GGT	Gamma-glutamyl transferase
NDR	National Diabetes Register
NPR	National Patient Register
NPDR	National Prescribed Drug Register
PC	Pancreatic cancer
TC	Total cholesterol
TG	Triglycerides
